# Pro-senescence therapy for hepatocellular carcinoma

**DOI:** 10.18632/aging.100601

**Published:** 2013-09-18

**Authors:** Susanne Muehlich, Thomas Gudermann

**Affiliations:** Walther Straub Institute of Pharmacology and Toxicology, Ludwig-Maximilians-University Munich, Germany

**Figure 1:**
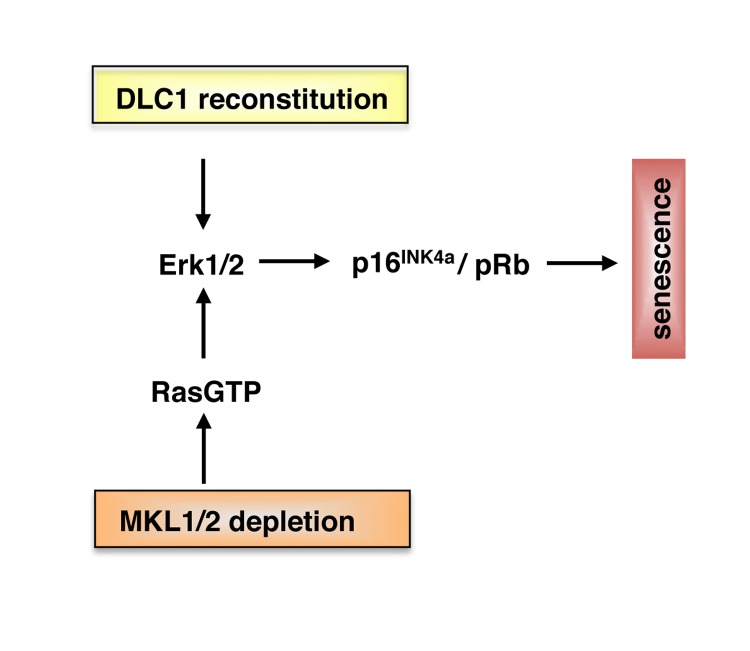
Model for senescence induction in HCC with DLC1 loss Reconstitution of DLC1 and depletion of MKL1 and 2 induce cellular senescence via activation of MAPK and p16INK4a/pRb pathways (see text for details).

Hepatocellular carcinoma (HCC) is one of the most frequent human malignancies with poor prognosis. Due to the current limitations of therapeutic options, there is a pressing need to elucidate the molecular mechanisms underlying human hepatocarcinogenesis and to identify novel targets for systemic therapy. It has recently been shown that Deleted in Liver Cancer 1 (DLC1), encoding a RhoGAP (Rho GTPase activating protein), is a tumor suppressor whose allele is lost in ~ 50% of liver, breast, lung and 70% of colon cancers [1, 2]. Identification of the relevant signaling pathways initiated by DLC1 loss may therefore allow for a more specific and personalized therapy of HCC. Our earlier work showed that DLC1 loss results in constitutive nuclear localization of Megakaryoblastic Leukemia 1 and 2 (MKL1 and 2) proteins, which are coactivators of the transcription factor Serum Response Factor (SRF) governing fundamental biological processes such as cell migration, cell growth, differentiation and cytoskeletal organization [3, 4]. A key feature of MKL1/2 regulation is that the proteins reside in the cytoplasm in an inactive conformation and translocate into the nucleus upon serum stimulation and actin polymerization [5, 6]. MKL1 nuclear localization following DLC1 loss is brought about by activation of the RhoA/actin signaling pathway and impairment of MKL1 phosphorylation, resulting in constitutive activation of tumor-relevant MKL1/2 target genes and enhanced HCC cell proliferation [4]. These findings led us to test whether depletion of MKL1/2 could block the proliferation of DLC1-deficient HCC cells. Indeed, DLC1-deficient HCC cells cease to grow in response to MKL1/2 depletion and display characteristic senescence-associated features including flat morphology, G1 arrest and induction of senescence-associated beta-galactosidase activity [7]. The MKL-knockdown mediated senescence response is caused by activation of the oncogene Ras and results in elevated p16^INK4a^ expression, hypophosphorylation of the retinoblastoma (Rb) protein and upregulation of components of the senescence-messaging secretome. The same repertoire of oncogene-induced senescence factors is induced upon DLC1 reconstitution, suggesting that DLC1 exerts its tumor suppressive effects via engagement of the same key effector pathways [7] (Figure 1). To assess the in vivo relevance for our observations, we employed polyethylenimine (PEI) complexation as an efficient tool for in vivo siRNA delivery into athymic nude mice bearing HCC tumor xenografts. Depletion of MKL1/2, as well as MKL1 alone completely abolishes tumor growth. Moreover, the regression of HCC xenografts is associated with oncogene-induced senescence [7]. This study raises a number of important questions. First, how does MKL1/2 depletion lead to Ras activation? Does MKL1/2 engage a RasGAP (RasGTPase activating protein) that causes senescence by activation of endogenous Ras and Ras effector pathways? Likewise, activation of one of the nine RasGEF (RasGTPase guanine nucleotide exchange factors) catalyzing the exchange of Ras-GDP for Ras-GTP could lead to hyperactivation of Ras. At present, it is not known which of the myriad of MKL1/2 target genes affects the senescence response (Figure 1). This will be a challenging task to tackle in the future. Second, why is MKL1 knockdown alone sufficient to curb HCC xenograft growth? It should be noted that inhibition of Rho-actin-dependent signalling to SRF in HeLa, NIH3T3 and MDA-MB-231 cells requires ablation of both MKL1 and MKL2 [3, 8]. Our work suggests that MKL1 depletion is sufficient to suppress HCC-relevant MKL1/2 target genes. Additional experiments are needed to identify the MKL target genes driving hepatocarcinogenesis. Third, which mechanisms are employed by MKL1/2 to evade senescence?

Interestingly, senescence pathways can be re-engaged upon depletion of MKL1/2 or reintroduction of DLC1 (Figure 1). Given the efficacy of MKL1/2 downregulation by PEI complexes, a therapeutic strategy to induce senescence by antagonizing MKL1/2 can be utilized to combat HCC. Such a strategy gains even more impetus since tumor suppressors such as DLC1 are generally not amenable to direct therapeutic targeting and blocking of Rho by geranylgeranyltransferase inhibition appears to be a suboptimal option due to the widespread cellular distribution of geranylgeranylation of proteins. Identifying MKL1/2 inhibitors will therefore be a promising goal for the future.
